# Diet and Genetics Influence Beef Cattle Performance and Meat Quality Characteristics

**DOI:** 10.3390/foods8120648

**Published:** 2019-12-06

**Authors:** Felista W. Mwangi, Edward Charmley, Christopher P. Gardiner, Bunmi S. Malau-Aduli, Robert T. Kinobe, Aduli E. O. Malau-Aduli

**Affiliations:** 1 Animal Genetics and Nutrition, Veterinary Sciences Discipline, College of Public Health, Medical and Veterinary Sciences, Division of Tropical Health and Medicine, James Cook University, Townsville, QLD 4811, Australia; felista.mwangi@my.jcu.edu.au (F.W.M.); christopher.gardiner@jcu.edu.au (C.P.G.); robert.kinobe@jcu.edu.au (R.T.K.); 2 CSIRO Agriculture and Food, Private Mail Bag Aitkenvale, Australian Tropical Sciences and Innovation Precinct, James Cook University, Townsville, QLD 4811, Australia; Ed.Charmley@csiro.au; 3 College of Medicine and Dentistry, Division of Tropical Health and Medicine, James Cook University, Townsville, QLD 4811, Australia; bunmi.malauaduli@jcu.edu.au

**Keywords:** diet, genetics, meat quality characteristics, tropical beef cattle, stearoyl-CoA desaturase, fatty acid binding protein 4, fatty acid synthase, *Desmanthus* legumes, supplementation, growth performance

## Abstract

A comprehensive review of the impact of tropical pasture grazing, nutritional supplementation during feedlot finishing and fat metabolism-related genes on beef cattle performance and meat-eating traits is presented. Grazing beef cattle on low quality tropical forages with less than 5.6% crude protein, 10% soluble starches and 55% digestibility experience liveweight loss. However, backgrounding beef cattle on high quality leguminous forages and feedlot finishing on high-energy diets increase meat flavour, tenderness and juiciness due to improved intramuscular fat deposition and enhanced mono- and polyunsaturated fatty acids. This paper also reviews the roles of stearoyl-CoA desaturase, fatty acid binding protein 4 and fatty acid synthase genes and correlations with meat traits. The review argues that backgrounding of beef cattle on *Desmanthus*, an environmentally well-adapted and vigorous tropical legume that can persistently survive under harsh tropical and subtropical conditions, has the potential to improve animal performance. It also identifies existing knowledge gaps and research opportunities in nutrition-genetics interactions aimed at a greater understanding of grazing nutrition, feedlot finishing performance, and carcass traits of northern Australian tropical beef cattle to enable red meat industry players to work on marbling, juiciness, tenderness and overall meat-eating characteristics.

## 1. Introduction

Beef plays a significant role in global human nutrition. It is the third most consumed meat in the world after poultry and pork at 6.4, 14.0 and 12.2 kg per capita, respectively [1]. Beef consumption continues to rise in line with growth and increase in population and household incomes. By 2027, it is estimated that beef consumption will be 8% and 21% higher in the developed and developing countries, respectively, compared to the 2015–2017 average [2]. Beef is a nutrient-dense food that provides health-beneficial macro- and micro-nutrients for humans. A 100 g serving of beef provides more than the 25% recommended dietary intake (RDI) of protein, niacin, vitamin B_6_, vitamin B_12_, zinc and selenium, and more than 10% RDI of phosphorus, iron and riboflavin. Beef protein is of certain characteristics and contains all the essential amino acids [3] and provides antioxidants such as carnosine and anserine [4,5]. 

On the world stage, Australia is among the major global meat industry players in terms of beef production and exports. In 2018, Australia was ranked seventh in world beef production and third in beef exports after Brazil and United States at 2.1, 1.6, and 1.5 million tons of carcass weight equivalents (CWE), respectively [6]. The beef cattle industry contributes significantly to the Australian economy, accounting for 20% ($12.1 billion) of the 2016–2017 total gross value of farm production and 22% of the total value of export income. The Australian beef cattle population is currently 23 million head and occupies about half of Australian farms and 75% of the total agricultural land mass [7]. Half of the national beef cattle herd is in northern Australia with 43% in Queensland and 16% in Western Australia and Northern Territory [8]. Queensland alone accounted for 1.1% of the global beef herd in 2017 and 8% of world beef exports in 2016 [9]. The major breeds in northern Australia are Brahman, Santa Gertrudis and Droughtmaster; all bred for tick resistance and heat tolerance, but their meat is comparatively different to temperate breeds [10]. To increase productivity and meat characteristics, these cattle breeds are sometimes crossed with *Bos taurus* to maintain at least 5/8 *Bos indicus* genetic composition to ensure adequate heat tolerance and tick resistance [9,10]. Several composite breeds consisting of half *Bos taurus* and *Bos indicus* such as Belmont Red, NAPCO Composite and AACO have been developed by crossing the Brahman with British, European and African breeds [11]. However, the challenge of low pasture quality and quantity remains a major limitation to beef production [9,12] and this is where the use of nutritional supplementation with *Desmanthus*, an environmentally well-adapted and vigorous tropical legume that can persistently survive under harsh tropical conditions, has the potential to drive animal performance, and improve meat characteristics. 

Meat characteristics is the culmination of the acceptability of a meat product in relations to its colour, intramuscular fat content, healthy fatty acid (FA) composition, tenderness, juiciness, flavour and aroma. Increasingly, market demands for meat products with healthy nutritional attributes and overall sensory characteristics are key factors strongly influencing willingness-to-pay decisions of beef consumers. Wolcott et al. [13] reported the findings of a broad consumer taste panel assessment of beef from cattle of various genetic, nutritional and environmental backgrounds in Australia, which demonstrated a measurable and negative impact of *Bos indicus* content on meat characteristics traits of tenderness, marbling and juiciness. FA composition of ruminant muscle tissues is essential to meat-eating characteristics due to its influence on flavour and tenderness, and published results suggest it is controlled by genetic factors such as genes responsible for lipids synthesis and metabolism [14]. Omega-3 long-chain polyunsaturated fatty acids (n-3 LC-PUFA) are beneficial in improving brain and retinal development, maternal and offspring health, cognitive function and psychological status in humans [15]. Delta-5 (Δ5), Δ6 and Δ9 desaturases are crucial enzymes in polyunsaturated fatty acids (PUFA) metabolism, and their activity can be influenced by several factors like dietary fatty acids and type of biological tissue [16]. Fatty acid binding proteins (FABPs) are conserved intracellular lipid-binding proteins that bind FA and other lipids reversibly. Fatty acid binding protein 4 (FABP4) is among the nine identified tissue-specific cytoplasmic FABPs [17]. FABP4 gene is expressed in the adipose tissue and plays an essential role in lipid metabolism and homeostasis. It interacts with peroxisome proliferator-activated receptors, binds to hormone-sensitive lipase [18] and is an essential candidate gene affecting intramuscular fat deposition. However, FABP4’s association with fatness traits in cattle varies from one study to another [19]. For example, FABP4 gene polymorphisms were significantly associated with backfat thickness [20], marbling and carcass weight [21] in Korean Hanwoo cattle. In contrast, FABP4 was associated with palmitoleic acid only in Japanese Black cattle [22]. Other genetic determinants of meat characteristics traits include the stearoyl-coA desaturase (SCD) and fatty acid synthase (FASN) genes. 

The primary aim of this review was to explore the published literature reporting the effects of nutritional grazing, dietary supplementation and roles of SCD, FABP4 and FASN genes on beef cattle performance and subsequent carcass and meat characteristics. The review also identifies current knowledge gaps that could underpin future research in nutrition-genetics interactions aimed at a greater understanding of grazing nutrition, feedlot finishing performance and carcass traits with a focus on tropical northern Australian beef cattle and the effect on marbling, juiciness, tenderness and overall meat-eating characteristics. 

## 2. Tropical Northern Australian Pastures and Beef Production

Beef production in northern Australia is heavily dependent on extensive tropical pasture grazing systems [23] of mainly native pastures dominated by C4 grasses [24]. In the northern rangelands, pastures are mainly unimproved with limited use of exotic pasture species in some regions of Queensland [25]. Black Speargrass (*Heteropogon contortus*) and *Aristida-Bothriochloa* grasslands dominate the more productive areas of eastern Queensland. In northern and western Queensland, Northern Territory and Western Australia Mitchell grass (Astrebla spp.), perennial tallgrass and shortgrass grass species, and spinifex (Triodia spp.) dominate [25,26]. *Stylosanthes* legumes are widely sown across northern Australia’s light textured soils to improve pasture nutritive value [27]. On the contrary, there was lack of suitable legume pasture for clay soils until recently [28]. Clay soil typify much of northern Australia pasture land [29]. For example, Vertosol (cracking clay) soils occupy 28% of Queensland’s total area, and are associated with grasslands, eucalypt woodland and brigalow/gidgee forests [30]. The most predominant pastures in these clay soils are *Asterbla* spp (Mitchell grasses) and *Iseilema* spp (Flinders grasses) with few sown pastures such as *Cenchrus ciliaris* (Buffel grass) in the Brigalow belt [31]. With the exception of young leaves and seeds, native pastures are of relatively low nutritional value at the end of summer growing season. During winter, growth is limited by temperature [32] and most native pastures are susceptible to frost leading to rapid decline in nutrient value [33,34]. 

The pastures are highly seasonal with growth occurring in the wet season (November to April), and ceases in 4 to 7 months of the year when conditions are too dry and/or too cold [35,36]. During the transition period from rainy to dry season, pastures decline in leaf to stem ratio caused by over 50% loss in leaf mass, crude protein content drops below 8% and the proportion of dead material increases, thus rendering the pastures less nutritionally beneficial and less palatable to cattle [37,38]. In addition, pastures deteriorate after several years of grazing due to nitrogen run-down stress [29]. The resulting poor nutrition leads to poor reproductive performance, slow growth rate, loss of body condition, increased susceptibility to parasites and diseases, increased turn-off age [35,36,39] and increased enteric methane emissions [40]. 

Forage quality is determined by nutrient concentration, intake, nutrient availability, and partitioning of metabolized products within animals [41]. Low quality forages contain less than 10% soluble sugars and starches, crude protein is below 8% and digestibility less than 55%. Utilization of these forages is limited by low intake due to physical fill limits and slow digestion as a result of high cell wall content and minimal nutrients available to support an efficient rumen microbial growth [38,41]. A study summary of data from 11 studies depicted a linear relationship between forage crude protein content and liveweight gain in cattle. Forage CP below 5.6% resulted in weight loss of up to 6 g/kg MBW (metabolic body weight), but above 5.6% resulted in 5–27 g/kg MBW gain daily [42].

### 2.1. Beef Cattle Responses to Under-Nutrition

Beef cattle use their evolutionary adaptation mechanisms which are either short (days), medium (weeks) or long-term, to cope with periods of under-nutrition. Short-term adaptations are in response to diurnal feeding frequency or daily changes in feed intake; mid-term changes appear within weeks of change; while long-term adaptations necessitate that the animals get into a new equilibrium involving different nutritional and physiological changes [43].

#### 2.1.1. Decrease in Liveweight

Decrease in liveweight (LW) after short-term underfeeding takes place due to gut-fill variation amounting to 4–5 kg LW/kg decrease in DM intake. For instance, digesta in the reticulo-rumen of a fed ruminant animal weighs up to 15% of body weight [44]. Medium-term under nutrition leads to organ and tissue mass variation. Liver and digesta-free gastrointestinal track reduction of more than 50% was reported after three weeks of restricted dietary access to maintain body weight [45]. Mid- and long-term weight losses are due to decreases in portal and hepatic blood flows as well as mobilization of fat, muscle and bone tissues in the reverse order of how they were deposited. The latest maturing tissues are fairly more sensitive due to physiological priority [43,44]. 

#### 2.1.2. Metabolic and Body Composition Changes

**Figure 1:**
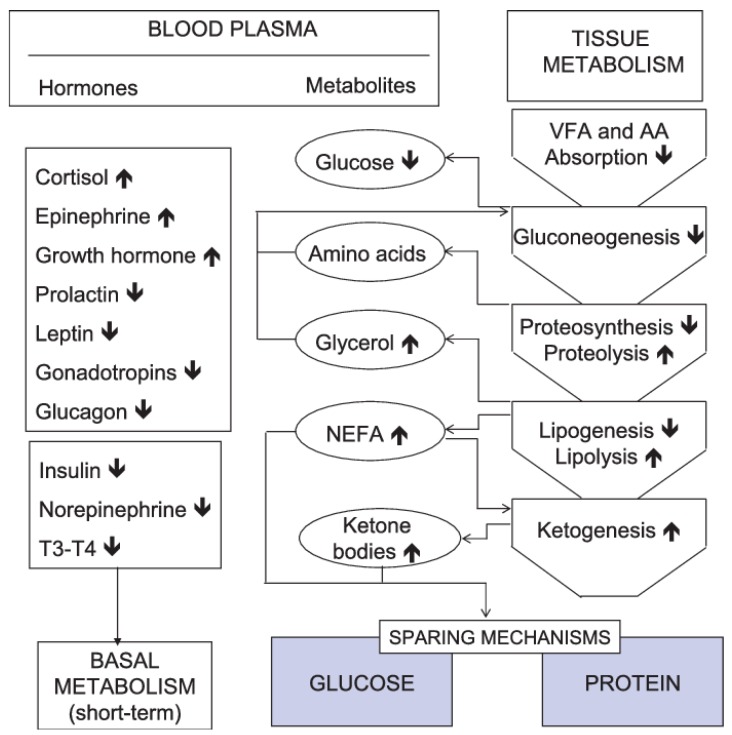
Metabolic and endocrine adaptations to undernutrition in the ruminant. NEFA: non-esterified fatty acids, VFA: volatile fatty-acids, AA: Amino-acids, T3-T4: thyroid hormones. Bold upward and downward pointing arrows indicate increase or decrease in tissue metabolism, blood plasma hormone or metabolite levels, respectively [43].

Storage triglycerides in the adipose tissue are hydrolysed to release FA which are oxidized directly to energy and broken down into ketone bodies (aceto-acetate, hydroxy-butyrate and acetones) in the liver [44]. The liver also incorporates FA into lipoproteins and triacylglycerols in the blood. Ketone bodies, lipoproteins and triacylglycerols act as sources of energy in peripheral tissues (Figure 1) [44]. Mobilized FA from the adipose tissue results in elevation of blood non-esterified fatty acids (NEFA). The liver removes 10% of NEFA from the blood during each cycle pass and converts half of all NEFA into ketone bodies. Hence, an increase in NEFA results in an increase in blood ketone bodies [40].

In periods of undernutrition, gluconeogenesis from propionate decreases due to a decrease in propionate availability. This is partially compensated by gluconeogenesis from amino acid (AA) proteolysis, glycerol lipolysis and lactate recycling. These metabolic changes are controlled by teleophoretic hormones such as insulin, glucagon and norepinephrine and together with decreased splanchnic tissues mass and variation in body composition, result in reduced energy expenditure. Mid-term experiments (several weeks) showed that portal-drained viscera, liver and skeletal muscles contributed to changes in energy expenditure of 17–61%, 14–44% and 5–7%, respectively [43,46]. These responses reduced growth rate and resultant beef since carcass fat content is a major factor that defines meat characteristics parameters like texture and taste [47,48].

### 2.2. Nutritional Supplementation to Improve Beef Cattle Performance on Low Quality Pastures

#### 2.2.1. Feed Supplements During Grazing

Numerous studies (Table 1) indicate that high feed conversion efficiencies and medium to high levels of production can be achieved by ruminants fed poor-quality tropical forages that are adequately supplemented with critical nutrients [49]. Metabolizable energy utilization efficiency of a forage can exceed that of grain-based diets when supplemented appropriately. The supplements optimize availability of nutrients for rumen fermentative digestion and utilization of nutrients that are products of fermentation [38]. Batista and colleagues [46] observed that supplements with a high proportion of rumen degradable protein, favour nitrogen recycling and promote increased microbial protein synthesis in beef cattle. Supplementation of the drinking water of steers fed Pangola grass (*Digitaria eriantha*) hay with Spirulina was found to increase ammonia-N concentration, propionate and branched- chain fatty acids in the rumen fluid. However, this study did not observe any positive effect of Spirulina supplementation on steer liveweight gains [50]. Non-protein N supplements are also supplied together with molasses to provide readily available energy for rumen microbes to synthesize microbial protein [42].

Supplements are reported to stimulate feed intake and liveweight gain (Table 1) to achieve up to one kg daily [56]. Supplementing cattle with urea together with molasses or other readily available energy sources at 2.8% N increases forage intake and prevents liveweight loss [42]. However, the cost of supplementation during grazing in an extensive grazing system is a limiting factor, hence it is mainly used for weaners and the breeding herd or for whole herd survival [56,57].

#### 2.2.2. Augmenting Pastures with Legumes

Incorporating a highly digestible forage into low digestible pastures supplies vitamin A, essential minerals, ammonia, peptides and amino acids. It also provides a highly colonized fibre source to ‘seed’ bacteria on the less-digestible fibre, thus improving total digestibility [38,58].

*Nutritional benefits:* Many studies have recognized the potential of legume pastures to improve beef cattle production in the tropics [36,39,59]. Legumes are rich in protein compared to tropical grasses due to the different biochemical pathways of carbon fixation during photosynthesis [60]. The protein therefrom can avail a renewable protein source to cattle grazing low quality grass pastures at a low cost [61]. Although the growth rate of cattle under grass only, or grass-legume pasture combination is similar early in the growing season, as the season progresses, legume-grass pasture-fed cattle gain more weight than grass-only fed cattle [62]. This is due to slower nitrogen decline in legumes as compared to all-grass pastures that leads to higher nutrition value of mature or dry legume pastures [36]. The introduction of legumes on grass-based pastures improves animal energy and protein intake, feed conversion and rumen function, as well as increases mineral and vitamin availabilities [39,63]. Steers grazed on *Leucaena leucocephala* and *Urochloa brizantha* pastures had higher weight gains compared to those on *Urochloa brizantha* only pasture [64]. 

*Effect on rumen lipid and protein degradation:* Some pasture legumes are known to produce tannins [65]. Moderate levels of tannins (<50 g/kg DM) reduce protein degradation in the rumen without depressing rumen fibre digestion or voluntary feed intake. At 20–40 g/kg DM, tannins may bind to the dietary proteins during mastication, thereby shielding them from microbial degradation [66,67]. This increases outflow of dietary protein to the duodenum and protein digestion and absorption in the small intestines [68,69].

**Figure 2:**
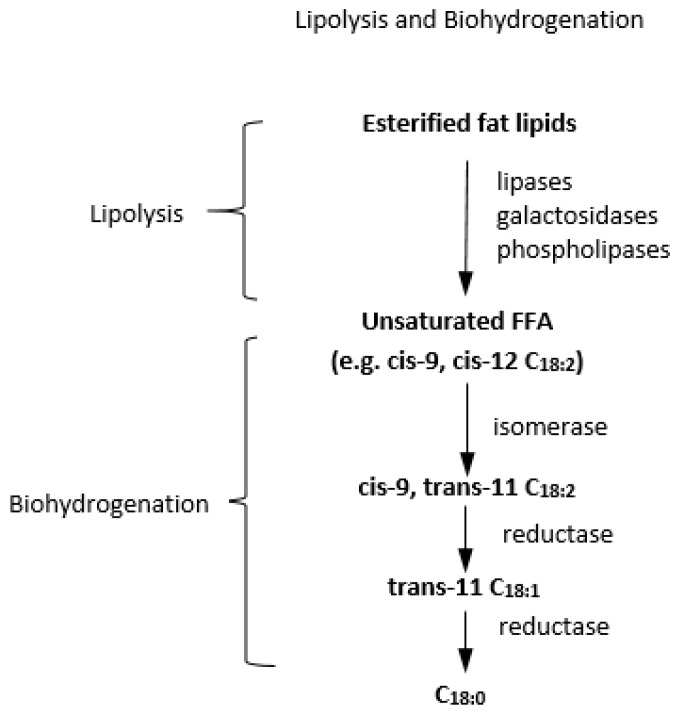
Key steps in lipolysis and biohydrogenation to convert esterified fatty acids to saturated fatty acids in the rumen. FFA: free fatty acids [71].

Tannins may inhibit or slow down lipid biohydrogenation (BH) in the rumen [70]. Dietary herbage lipid composition is made up of membrane lipids: glycolipids and phospholipids, while seed lipids are polar lipids, mainly triacylglycerides [71]. After ingestion, dietary triglycerides and phospholipids are hydrolysed into glycerol, FA and small amounts of mono- and diglycerides by microbial lipases in the rumen. Glycerol undergoes rapid fermentation to yield propionic acid as the major product [72], while unsaturated FA are hydrogenated into saturated trans FA by microbes [44,71]. Biohydrogenation involves isomerization of cis-12 double bonds in unsaturated FA to a trans-ll isomer, followed by hydrogenation of the cis-9 bond in linoleic acid by microbial reductase into saturated FA (Figure 2) [71].

In an in vitro study, tannin extract from *Schinopsis lorentzii* reduced biohydrogenation of α-linolenic acid (ALA) from 43% to only 13% in flaxseed diet after 24 h incubation [73]. Tannin-containing forage (Sainfoin) had no effect on ALA BH, but diets containing tannin extracts (7.9% of dietary DM) reduced BH by 20% in vitro [74]. Incubating hay and concentrate diet in vitro at 1.0mg/ml of cow buffered ruminal fluid increased vaccenic acid and reduced stearic acid concentration by 23 and 16%, respectively, compared to the control. Tannin was extracted from *Ceratonia siliqua*, *Acacia cyanophylla* and *Schinopsis lorentzii* [75]. Feeding lambs with *Cistus ladanifer* at 200g/kg DM reduced complete rumen BH by 36% in lambs, but had no effect when fed at 50g/kg [76]. The inclusion of Sainfoin in a Timothy grass silage diet of lambs increased the accumulation of ALA in rumen digesta [77]. Protecting unsaturated FA from biohydrogenation in the rumen increases the levels of unsaturated FA absorbed through the intestines into the blood stream. Plasma n-3 PUFA of steers infused sunflower oil into the duodenum was two-fold compared to control [78]. These studies were conducted in controlled environments and some used tannin extracts and/or included oil supplements in the feeds [74,76]. 

*Effect on parasite control:* Tannin-containing diets impose higher parasite tolerance in different species of grazing animals [66,79]. Tannins exert their anti-parasitic effect by decreasing the viability of larvae, thus interfering with egg hatching and/or improved immunity as a result of improved protein nutrition from reduced rumen protein degradation [80]. In vitro and in vivo studies in small ruminants have reported significant effects of tannin extracts and tannin-containing legumes on faecal egg count, development of eggs to larvae and decreased larvae motility [81,82]. Naturally-infected lambs grazing chicory (*Cichorium intybus*) and birdsfoot trefoil were reported to have fewer helminth parasites than lambs grazing ryegrass or white clover. Birdsfoot trefoil grazing reduced fecal egg count significantly compared to all the other forages [83]. Red deer calves infected with deer-origin gastrointestinal nematodes and lungworm (*Dictyocaulus* sp.) larvae for five weeks were allocated into either lucerne (0.1% condensed tannins; CT), birdsfoot trefoil (1.9% CT) or sulla (*Hedysarum coronarium* L.) (3.5% CT) and slaughtered after seven weeks. Abomasal nematode burdens had significant negative linear relationships with dietary CT concentration, although no substantial differences were observed in faecal egg counts [84].

#### 2.2.3. Use of Legumes in Northern Australia

For the past six decades, the northern Australian beef industry has used tropical pasture legumes [85]. These legumes have been recognized as the best long-term alternative to increase grass pasture productivity. However, adoption level still remains low [86]. For a long time, most attention was directed at lighter textured soil pastures [87] and only little emphasis was placed on legumes adapted to dark clay soils, resulting in genotypes that were either not sufficiently productive or persistent [88,89,90]. However, attention has been directed to pastures of heavier textured soils of central and southern Queensland in recent years, leading to the development of more suitable perennial legume species and varieties such as *Stylosanthes seabrana* (Caatinga stylo), *Clitoria ternatea* (Butterfly pea), *Macroptilium bracteatum* (Burgundy bean) and *Desmanthus*. Inspite of this, only limited published literature on animal growth and performance from these legumes exists. Stylosanthes, Butterfly pea and *Desmanthus*-grass pastures were observed to improve weight gains compared to grass only pastures numerically throughout the year [59]. Steers grazed on Butterfly pea-grass and Caatinga stylo-grass pastures had no difference in weight gain compared to grass only pasture in the first year of establishment. However, 31 and 68 kg/ha difference, respectively, was observed over a five-year period [91]. 

*Desmanthus* has gained attention in recent years due to its palatability, high protein content, non-toxic characteristics, anti-methanogenesis demonstrated in vitro [92] and its ability to establish and persist well in clay soils [88,89,90]. Two varieties of *Desmanthus, D. virgatus and D. leptophyllus,* were found in old trial sites after 25 years of establishment on black cracking clay soil surviving droughts, floods, frost and commercial grazing [93]. *Desmanthus* is highly nutritious with at least 14% CP in whole plant and 22% in leaves [39,94]. *Desmanthus* is, therefore, a good legume choice for the clay soils of northern Australia [95,96]. Since 2012, 35,000 ha of *Desmanthus* have been established in northern Australia [97] and necessitates studies to be carried out to determine its effect on beef cattle performance and meat characteristics. A short term 90-day study, reported up to 40kg higher liveweight in steers grazed on *Desmanthus*–Buffel grass pasture than Buffel grass only [98]. Supplementing Rhodes grass fed goats with *Desmanthus* increased dry matter intake, liveweight gain, loin-eye muscle area and hot carcass weight significantly, compared to urea and cottonseed meal supplements [99]. Similarly, a 10-week study reported that *Desmanthus*–Mulato grass diet increased liveweight gain in goats significantly compared to those on Mulato grass only [100]. In contrast, growing goats demonstrated poor acceptance of *Desmanthus bicornutus* compared to Leucaena, alfalfa, and lablab, resulting in lower weight gains [39]. Gardiner (pers com) however has observed *D. bicornutus* in pasture to be very palatable to cattle and cv JCU-4 fed in metabolic chambers was well accepted.

### 2.3. Feedlot Finishing of Tropical Pasture-Backgrounded Cattle

Feedlot finishing is an important phase in the beef supply chain of pasture backgrounded beef cattle. In 2017, 50% of Queensland beef herds were finished in the feedlot with grains high in sugar and fat [9]. Lot-feeding helps to finish cattle during periods of limited pasture availability. This allows beef products to meet certain yardsticks of a wide range of markets, marketing of more even products, reduce farm-stocking pressure during the dry season and help to plan for the marketing season [101,102]. A comprehensive review by Drouillard and Kuhl [103] reported that diet characteristics during backgrounding affects cattle performance in the feedlot. Cattle grazed on poor pastures that restricted growth moderately led to complete compensatory growth during lot-feeding, while those backgrounded on poor pastures resulting to weight loss failed to achieve compensatory growth. Cattle grazed on endophyte-infected fescue were compared to those grazed on endophyte-infected fescue-clover mix and endophyte-free fescue. Cattle grazing on endophyte-infected fescue-clover mixture consistently performed best during grazing and finishing [104,105]. A meta-analysis of 20 dry-lot and 12 grazing studies showed that cattle fed high energy diets during backgrounding had lower final body weights than those grazing or fed on restricted energy. However, this study did not analyse the effect of dietary protein content [106].

## 3. Meat characteristics

The ultimate goal of the beef cattle industry is to provide consumers with beef that is safe and of high eating characteristics. The major determinant of meat characteristics is eating characteristics, influenced mainly by intramuscular fat content, low fat melting point, tenderness, juiciness and flavour [107]. Carcass fat deposition and meat FA composition both play important roles in eating characteristics variation [108,109,110]. Consumption of beef fat can help in the transport and absorption of fat-soluble vitamins and exerts positive effect on immune response [111]. 

Beef fat is primarily categorized into three; subcutaneous, intermuscular and intramuscular fat [112]. Saturated FA (SFA) whose levels are high in ruminant meat due to hydrogenation of dietary unsaturated FA in the rumen, are associated with health risks such as coronary heart disease [113,114], although this association remains controversial [115,116]. Monounsaturated FA (MUFA) is reported to be associated with lower mortality rate [117] although other studies did not find any association [115]. Increasing the level of n-3 polyunsaturated FA (PUFA) in the human diet is important to overcome the imbalance resulting from high consumption of plant oils rich in linoleic acid [118]. Long-chain n-3 and n-6 PUFA improve growth, brain and retinal development, maternal and offspring health, cognitive function and psychological status in humans [15]. Also, conjugated linoleic acid (CLA) and n-3 FA confer anti-inflammatory effects [119]. Recommendations for various dietary fat fractions are 15–35%, <10%, <2.5–9%, <2–3% and <1% of total energy intake from total fat, SFA, n-6 PUFA, n-3 PUFA and trans fatty acids, respectively [120,121] and ratios of PUFA:SFA at 0·45 and n-6:n-3 PUFA below 4 [122]. As a result, there is increasing focus on studies aimed at elevating the levels of beneficial n-3 LC-PUFA and reducing saturated fatty acids in beef, especially in intramuscular fat (IMF), commonly referred to as marbling, since it cannot be trimmed out [3,113]. 

Marbling is associated with carcass fatness. A positive correlation between total carcass fat content and subcutaneous fat thickness with marbling has been observed [123,124]. For instance, a genetic correlation of 0.91 between marbling score and muscle lipid content was reported [125]. Increase in subcutaneous fat thickness from below 0.19 mm to over 1.40 mm transitioned marbling score from ‘devoid’ to ‘abundant’ [126]. An increase in carcass fat content and subcutaneous fat thickness from 187 to 217 g/kg and 6.6 to 8.3 mm, respectively, increased marbling score from 2.2 to 2.6 in steers [127], while an increase in carcass fatness influenced FA composition and PUFA:SFA ratio [128]. Marbling fat consists of more unsaturated FA compared to other fats in beef, hence a higher PUFA:SFA ratio. It also contains more oleic acid and less stearic acid [3,129].

### 3.1. Effect of Intramuscular Fat on Beef-Eating Characteristics

#### 3.1.1. Tenderness

Variation in tenderness is attributed to animal age, pre- and post-slaughter carcass handling, post-mortem pH decline, genetic make-up and carcass composition, mainly marbling [108,130,131]. Subcutaneous and intermuscular fats provide insulation for muscles to prevent cold shortening. Muscles cool at a slower rate and rigour is attained at higher temperatures. Leaner lamb carcasses with lower marbling scores and less subcutaneous fat thickness were reported to be tougher than those with more fat [132,133]. Similarly, Jeremiah [126] reported a higher tenderness score for steers and heifers with higher subcutaneous fat thickness and marbling as scored by both trained and untrained panel of consumers. High marbling score as in Kobe beef that can exceed <200 mg/g fresh meat, cause dilution of fibrous proteins by soft fat, thus lowering the bulk density that may reduce resistance to shearing. Marbled fat cell expansion forces muscle bundles apart to result in opened up muscle structure [109,134]. Marbling fat concentration values above 30 mg/g muscle are suggested to result in optimum tenderness [48]. 

#### 3.1.2. Flavour

Animal nutrition status, diet, sex, breed and genetic make-up are factors that influence meat flavour [135]. Meaty flavour of cooked meat develops from a complex interaction of precursors from the fat and lean components of meat. Products of Maillard reactions between carbohydrates and proteins, such as pyrazines and thiazoles, and lipid degradation of aldehydes, alcohols and ketones, are the most important determinants of flavour [136,137]. Hence, meat composition plays an important role in flavour, which could explain the increase in flavour intensity with age in meat animals [108]. A trained panel reported higher flavour scores for beef from carcasses with higher subcutaneous fat thickness than those with minimal fat [126]. FA composition of the fat also plays a significant role in meat flavour. Linolenic acid was found to be positively correlated with milky-oily and sour flavour in beef, while oleic acid was negatively correlated [138]. Oleic acid is considered to be of major effect on the flavour of cooked beef [139]. FA oxidative degradation to form alkyl radicals occurs faster in PUFA than MUFA, while linolenic acid derivatives, eicosapentaenoic acid (EPA) and docosahexaenoic acid (DHA), are highly susceptible to oxidation giving rise to aldehydes [109].

Fats act as storage sites for skatoles and indoles, two compounds that play a significant role in meat flavour, but moreso in sheep than cattle. They are produced in the rumen through microbial deamination and decarboxylation of tryptophan. When they exceed the liver’s metabolism capacity, they are deposited in body fats, thus contributing to pastoral flavours in ruminant meat. At low levels, skatoles contribute to desirable odours and flavours, but at high levels, they produce a nauseating faecal odour [110,111]. Finishing grass-fed cattle with concentrate diet for at least 54 days reduces pastoral flavour significantly [112].

#### 3.1.3. Juiciness

Meat juiciness is the initial impression of moisture released on the meat surface during chewing and the degree of induced salivation [124]. Meat juiciness relies on water and fat contents, hence factors influencing water holding capacity and fat content of meat may influence juiciness [140]. Marbled fat provides lubrication between muscle fibres and increases the perception of juiciness by stimulating salivation while chewing [134]. Fat prevents drying out of meat during cooking [141]. However, some studies did not find any positive correlation between beef subcutaneous fat and marbling with juiciness [126].

### 3.2. Factors Influencing Beef Intramuscular Fat Content and Fatty Acid Composition

In beef, the lipid fraction generally contributes between 4–15% of carcass weight on fresh basis [142]. Out of these, four fifths are SFA, mainly composed of palmitic, stearic and oleic acid. The remaining fifth comprises 30 different FA [143]. In the intramuscular lipid fraction, values of 2–30% in the *Longissimus dorsi* muscle (LDM) has been reported [144]. 

Lipid fraction and FA composition are influenced by three major factors; namely, age of the animal, diet and breed. A study of fat content and FA profile in three breeds of cattle reported an increase in IMF and saturated FA percentage and a decrease in unsaturated FA of LDM with age [145]. In contrast, Jersey and Limousin cattle showed decrease in total SFA and increase in MUFA [146], while phospholipids showed a decrease in palmitate, stearate and oleate, but an increase in PUFA with age [147]. Age had no effect on Japanese Black cattle FA composition [148]. Beef cattle producers target early turn-off age of steers and heifers, usually below 28 months [149,150]. Details on mechanisms in which age influences beef fatty acids composition are not given as this is beyond the intended scope of this review.

Composition of backgrounding and finishing diets influence beef fatty acids profile. Differences are reported between cattle fed pasture versus concentrate diets [114], pastures containing varying plant secondary metabolites [151], and diets supplemented with oils [152], vitamins and minerals [153,154].

#### 3.2.1. Pasture Versus Concentrate Diets

Beef from pasture raised cattle contains higher levels of n-3 and MUFA compared to concentrate-fed cattle [114,155]. Tume [156] suggested that the effect is mainly attributable to individual ingredients in the diet and their combinations. Plants are the primary sources of n-3 PUFA due to their unique ability to synthesize ALA, which comprises at least half of the FA content of forages. ALA forms the building block of n-3 essential FA, and its elongation and desaturation results in the synthesis of EPA, DHA and docosahexaenoic acid (DPA) [113]. Moreover, biohydrogenation of unsaturated FA in the rumen is followed by microbial FA synthesis from dietary long-chain FA and de novo synthesis, amounting to 10–15% of bacterial dry mass that influences the FA profile of absorbed lipids [71,157]. Feeding Angus crossbred steers on forages and pasture only, increased muscle rumenic acid by four-folds compared to high-grain diet [158]. Continental crossbred steers fed on grass pasture had the highest intramuscular PUFA content and increasing dietary concentrate supplement led to a linear increase in SFA, increased n-6:n-3 PUFA ratio as well as a decrease in PUFA:SFA ratio [114]. Angus-cross steers backgrounded on pasture only were finished on corn-silage concentrate or pasture. Pasture finished steers had 61%, 21% and 22% less total fat, oleic acid and total MUFA compared to the concentrate group. Individual (linolenic acid, EPA, DPA, and DHA acids) and total n-3 FA concentrations and the ratio of n-6 to n-3 fatty acids were greater in forage than concentrate finished steers [159]. Grass-fed German Holstein and Simmental bulls had higher total PUFA and lower n-6-n3 ratio than concentrate fed bulls, but total SFA was similar [155]. Similarly, grass-fed Hereford steers had higher PUFA, lower MUFA and n6:n3 ratio than concentrate-fed steers, while SFA was not affected [160]. Fat deposition relies on consumption of surplus net energy [161], hence, grain feeding increases carcass total fat content due to high energy levels [124].

Time on feed also plays a significant role in beef FA composition. Steers raised on native range stocker operation were divided into eight groups, finished on a high concentrate diet and slaughtered serially at 28 days intervals from zero (control) to 196 days on a finishing diet. Carcass marbling score, subcutaneous fat thickness, LDM MUFA and total lipid percentage increased, while PUFA decreased with increase in days on concentrate diet. Differences in these parameters were observed after 112 days on the diet after which a plateau was reached [162]. These results should be interpreted with caution as age also affects carcass fat content and composition as well as marbling [163,164,165].

Some studies have reported the effect of pasture species on beef FA composition, while some reported no difference. For instance, alfalfa-finished steers had higher concentrations of linoleic acid and ALA than those finished on pearl millet and a combination of white clover, blue grass, orchard grass and tall fescue, but forage species did not affect total lipid content of the LDM in a 40-day study [159]. In a four-month study where steers grazed on tall fescue only, or combined with red clover or alfalfa, there was no effect of pasture on meat FA content [152]. Similarly, rib eye rolls from steers finished on tall fescue and meadow brome or birdsfoot trefoil for four months had similar marbling scores, n-6-n3 ratios and total SFA, MUFA and PUFA, but EPA was higher in birdsfoot trefoil finished steers [166]. The effect of pasture type on FA composition was reported in a 90-day study where lambs with access to shrubs produced meat with higher percentage of ALA, n-3, n-6, total PUFA and lower MUFA and n-6:n-3 ratio than those on grass only, but total SFA was similar [167]. The effect of different forage species may be due to plant secondary metabolites. Cattle grazing botanically diverse pastures with different plant secondary metabolites had higher intramuscular n-3 and total PUFA compared to cattle grazing predominantly ryegrass pastures with similar pasture FA profile [168]. Red clover reduces ruminal biohydrogenation of PUFA, possibly due to the protective effects of the polyphenol oxidase enzyme [169]. As discussed earlier, dietary tannin may inhibit or minimize rumen biohydrogenation of unsaturated FA and increase the level of n-3 PUFA in the blood circulation. LDM of lambs fed Sulla (*Hedysarum coronarium* L.) containing 1.8% condensed tannins had 24% more ALA compared to lambs fed Sulla and drenched with polyethylene glycol, a compound that binds and inactivates tannins [151]. *Desmanthus* contains up to 4.5% condensed tannins [92], hence grazing cattle on *Desmanthus* pastures may increase n-3 PUFA in beef.

#### 3.2.2. Oil Supplements

Dietary supplementation with n-3 LC-PUFA-rich oils has been shown to increase PUFA in the meat of ruminants [152] because at high concentrations, rumen microorganisms cannot hydrogenate these oils to any significant extent [170] and oil supplements also enhance *de novo* FA synthesis from their dietary precursors [118]. Steers supplemented with fish oil doubled the EPA and DHA contents in muscle phospholipids, while those supplemented with linseed increased the levels of ALA in muscle phospholipids from 9.5 to 19 mg/100 g and enhanced EPA synthesis from 10 to 15 mg/100 g in muscle with no effect on feed intake [118]. Lorenzen and colleagues [123] reported over 80% increase in CLA in beef from soybean oil supplementation compared to the control during finishing of steers. Soybean oil supplement increased CLA in the adipose tissue of steers [154]. Fish oil supplement increased n-3 LC-PUFA, including linolenic acid, EPA and DHA concentrations in the LDM of bulls and steers [153] and slightly increased the total FA in supplemented steers compared to the control [118]. Diet-protected fish oil and free fish oil increased total muscle EPA and DHA from 13 to 19 mg/100 g and 3 to 12 mg/100 g, respectively [171]. Effect of oil on beef FA composition is not unique to pure oil supplements. British x Continental crossbred steers were fed grass hay or red clover silage only or supplemented with either sunflower-seed or flaxseed concentrates to provide 5.4% oil in diet DM basis. Sunflower-seed or flaxseed supplements increased vaccenic, rumenic and n−6 FA in the *Longissimus thoracis* muscle significantly. ALA was over two-fold in flaxseed compared to sunflower-seed supplemented steers [142].

#### 3.2.3. Micronutrients

*Vitamin A:* Vitamin A or β-carotene deficiency results in elevated IMF content. Angus steers were fed low β-carotene and vitamin A cereal-based ration for 308 days with or without Vit A supplementation before slaughter. Supplemented steers scored 19% less marbling and the LDM IMF content was 35% lower than the control [172]. Supplementing Japanese Black cattle with vit A after 15 months of age reduced marbling score significantly. A correlation of −0.38 was observed between marbling and serum vit A just before slaughter [173]. Effect of vit A is proposed to be due to its derivative retinoic acid that restricts hyperplasia and/or by regulating the growth hormone gene resulting in a decrease in fat deposition [125]. Trans-retinoic acid, a metabolite of retinol, subdues differentiation of preadipocytes by suppressing the expression of peroxisome proliferator-activated receptor gamma (PPARγ) gene [174,175].

*Vitamin C:* Domestic animals normally do not receive dietary Vitamin C supplementation due to their ability to synthesize the vitamin in the liver [176]. However, plasma Vitamin C levels in beef cattle drop below the normal 2.4–4.7 mg/L range during the late fattening period, showing that Vitamin C plays an important role in adipogenesis [177]. Supplemented Japanese Black cattle receiving high-concentrate diets with Vitamin C during the late [178] or from middle fattening stage produced fatter carcasses with higher marbling scores than the control [177]. Increased lipogenesis is as a result of the positive effect of Vitamin C on adipocyte differentiation [179].

*Vitamin D and Calcium:* 1α,25-dihydroxyvitamin D_3_, the biologically active form of Vitamin D, inhibits the differentiation of preadipocytes through direct suppression of PPARγ protein [174]. Since 1α,25-dihydroxyvitamin D_3_ is critical for calcium homeostasis, low dietary intake of calcium leads to increased plasma 1α,25-dihydroxyvitamin D_3_ that suppresses adipocyte differentiation and reduces marbling [177]. Feedlot cattle with low plasma 1α,25-dihydroxyvitamin D_3_ levels had higher marbling scores than those with higher levels [180]. In contrast, high marbling scores were reported in Hanwoo steers finished on low calcium diets leading to high levels of plasma 1α,25-dihydroxyvitamin D_3_ than in steers finished on high calcium diets [181]. 1α,25-dihydroxyvitamin D_3_ may exert two contrasting functions on adipogenesis; inhibit adipocyte differentiation and promote fat accumulation in adipocytes, depending on the animals’ stage of growth [177].

#### 3.2.4. Cattle Breed

Studies have reported variation in beef fat deposition and FA composition due to genetic differences between cattle. Beef breeds differ from milk breeds and such differences are well documented. Generally, meat type breeds are able to deposit more fat than milk breeds. FA synthesis was reported to be higher in beef cattle subcutaneous and perirenal adipose tissues than in same tissues from dairy cattle of similar age and weight [182]. Differences between Jersey and Limousin [146,147], Japanese Black and Holstein [183] have been reported. German Holstein bulls had higher SFA and total PUFA compared to German Simmental bulls on similar diets, but breed had no effect on n-3 FA [184]. Nuernberg and colleagues [155] reported higher n-3, n-6, n-6:n-3 ratio and total PUFA in German Simmental beef bulls than in German Holstein bulls, but SFA levels were similar. CLA isomer cis-9, trans-11 concentration was higher in the LDM of German Holstein than German Simmental bulls. In a 24-month study, Galloway, White–Blue Belgian and German Holstein bulls were fed on the same diet and slaughtered at different ages between zero and 24 months. Carcass subcutaneous fat and LDM intramuscular fat were highest in German Holstein and least in White-Blue Belgian. At birth, stearic acid, oleic acid and n-3 FA were highest in the LDM of Galloway, while total unsaturated FA, PUFA and n-6 FA were highest in White-Blue Belgian. A similar profile was observed at 18 months of age except in n-3 FA that were similar in Galloway and WBB, but lower in German Holstein [145]. Comparing the FA profile of Japanese Black and Holstein steers subcutaneous neutral lipids showed lower myristic acid, palmitic acid, stearic acid and total SFA, but higher oleic acid, total MUFA and MUFA:SFA ratio in the Japanese Black than Holstein steers. However, intramuscular phospholipid FA profile was not affected except for palmitic acid [183]. Simmental and Red Angus steers at similar back fat finished level of 10 mm were compared for LDM fat profile. Total lipids, myristoleic acid, palmitoleic acid, vaccenic acid, along with n-6:n-3 ratio, were greater while EPA and total n-3 PUFA were lower in Simmental than Red Angus steers. Time on grain diet was a confounding effect in this study as the Angus spent 70 days less on the grain diet and were slaughtered 73 days younger than the Simental steers [185]. 

Sires may influence the FA content of their offspring. Japanese Black Wagyu cattle sired by different bulls were reported to have significantly different SFA and MUFA contents [186]. Heritabilities of FA and other carcass traits were reported to range from 14 to 36% in crossbred cattle [187]. These breed variances are probably due to differences in the activities of enzymes influencing gene expression and/or enzyme function [113]. The activity of Δ9-desaturase enzyme to convert palmitic to palmitoleic acid was observed to be greater in Simmental than Red Angus lipids [185]. 

### 3.3. Genes that Influence Carcass Fat Content and Fatty Acid Profiles

Several genes are reported to be responsible for variation in fat content and FA composition in beef. The genes encode for cocaine- and amphetamine-regulated transcript [188], leptin [189], diacylglycerol O-acyltransferase, the growth hormone 1 [190] sterol regulatory element-binding protein 1 [191], fatty acid synthase, stearoyl-CoA desaturase and fatty acid binding protein 4 [112,192,193,194]. This review will focus on fatty acid synthase, stearoyl-CoA desaturase and fatty acid binding protein 4 genes. 

#### 3.3.1. Stearoyl-CoA Desaturase (SCD)

SCD gene encodes for Δ9 desaturase enzyme and introduces a single double bond in SFA to convert them to MUFA. For instance, the enzyme desaturates stearic acid to oleic acid and trans-vaccenic acid. High concentration of oleic acid in beef is associated with soft fat and overall palatability in Wagyu and Hanwoo cattle. As a result, high activity of Δ9 desaturase enzyme is associated with soft fat in beef [144,195]. SCD catalytic activity is about twice higher in bovine marbled muscle tissue than in the subcutaneous adipose tissue. This agrees with higher MUFA levels observed in the muscle than subcutaneous adipose tissue [196,197,198]. SCD gene expression and activity is reported to increase after weaning [199] and preceding lipid filling in preadipocytes. Similarly, a gradual increase in de novo FA biosynthesis is observed after weaning, indicating that SCD activity is required for lipogenic activity in the subcutaneous adipose tissue to develop [144]. In another study, subcutaneous adipose tissue samples were collected from carcasses of pasture and feedlot cattle fed for 100, 200 and 300 days. Pasture-fed cattle adipose tissue had lower total SFA and higher total UFA than in feedlot cattle. Δ9 desaturase activity was much higher in pasture-fed than feedlot cattle [47].

SCD gene expression varies between and within breeds. Full-length bovine SCD cDNA from 20 Japanese Black steers was compared. Two types of SCD genes with single nucleotide polymorphisms (SNPs) in the open reading frame where valine (V) was replaced by alanine (A) were observed. The two SCD genes were genotyped and classified in 1003 Japanese Black carcasses into VV, VA and AA genotypes. Comparison of FA composition from the carcasses showed that SCD type A gene was associated with higher percentage of MUFA with 0.8% effect and lower IMF melting point. They concluded that SCD is one of the causes of genotype variation [200]. In contrast, SCD (878C>T) SNP was observed to have no association with FA profile in upper sirloin cuts of Aberdeen Angus and Blonde d’Aquitaine cattle [112].

#### 3.3.2. Fatty Acid Synthase (FASN)

FASN gene is abundantly expressed in the adipose tissue and encodes for fatty acid synthase, an enzyme that regulates the biosynthesis of long chain fatty acids. The enzyme plays a central role in de novo lipogenesis by catalysing all the reaction steps to convert acetyl-CoA and malonyl-CoA to palmitate. Association of FASN expression or polymorphisms with fat metabolism and obesity traits in cattle has been reported [188,201]. Analysing polymorphisms in thioesterase domain of FASN gene, which regulates the termination of FA synthesis, in Hanwoo cattle showed a significant association between g.17924G > A SNP genotypes with palmitic and oleic acid concentrations. For instance, GG genotype had 3.2% and 2.8% higher oleic acid concentration than the AA and AG genotypes, respectively. However, they did not observe any significant association between g.17924A > G genotypes and other examined FA such as myristic, stearic and linoleic acids [191]. GG genotype of g.17924A > G SNP was reported to result in higher UFA and fairly lower amounts of SFA than the AG and AA genotypes in other studies [192,202]. Another study was carried out to determine exonic SNPs in the gene encoding FASN with FA composition in Korean cattle. It was found that all the SNPs (g.12870 T > C, g.13126 T > C, g.15532 C > A, g.16907 T > C and g.17924 G > A), were associated with varying FA compositions and marbling. Genotypes CC, TT, AA, TT, and GG were associated with higher MUFA and lower SFA [203]. 

Some studies reported no relationship between FASN gene with fat thickness and marbling score. However, a significant relationship of the fat with DNA-protein kinase, known to play a role in transcriptional activation of FASN, was reported [204,205].

#### 3.3.3. Fatty Acid Binding Protein 4 (FABP4)

FABP4 is a gene highly expressed in the adipose tissue and encodes for fatty acid binding protein 4 that belongs to a group of FABPs. These binding proteins play a significant role in absorption, transport and metabolism of FA, and glucose homeostasis by interacting with peroxisome proliferator-activated receptors [18,206]. SNP 7516G > C of FABP4 was analyzed for association with IMF profile of upper sirloin cuts in Aberdeen Angus and Blonde d’Aquitaine cattle. CC genotype in Angus cattle was 52% and 64% lower in Myristoleic acid, and 33% and 35% lower in linoleic acid than CG and GG, respectively. Blonde cattle CC genotype had higher arachidonic acid and EPA, but lower oleic acid and total SFA than the CG. The GG genotype was observed in only one bull [112]. g.7516G > C polymorphisms were analyzed for association with marbling score and subcutaneous fat depth in Wagyu x Limousin crosses. A positive relationship between CC genotype and lower marbling and fat depth was observed. GC genotype had the highest scores while GG was in-between [18]. FABP4 SNPs were also reported to have an association with back fat thickness in Korean Native cattle [20].

## 4. Conclusions and Future Research

Beef is a nutrient-dense food and remains an important dietary component in global human nutrition. In northern Australia, the beef industry contributes immensely to the economy as it accounts for over half of Australia’s beef exports. It relies heavily on native pastures that are highly seasonal and of low quality resulting in weight loss during the dry season and a high turn-off age. Although supplementing beef cattle with protein and energy diets improves weight gain, cost is limiting, hence supplementation is not an economical option in extensive grazing systems. However, nutrient-dense diets are used to finish most northern Australian beef cattle herds to produce a more even product that meets certain yardsticks of a wide range of markets. Legumes are known to improve pasture and livestock production at a lower cost. However, most legumes do not survive or persist in clay soils prevalent in northern Australia. In recent years, several persistent pasture legumes in clay soils have been developed and trialed. Of specific interest is *Desmanthus*, a highly palatable, high protein content, non-toxic tropical legume with potential to reduce enteric methane emissions. Few available studies indicate that *Desmanthus* can be used to improve pasture quality and subsequent beef cattle productivity in northern Australia.

- However, only limited peer-reviewed published literature is available on the effect of *Desmanthus* on beef cattle growth and performance. These studies were either conducted indoors or in small sized paddocks (except one in 250 ha paddock) which may not be replicated in normal commercial farm settings. Hence, there is need to conduct more studies under commercial farm settings to determine the suitability of grass–*Desmanthus* pastures in northern Australian beef cattle production system.
- Tannin-containing pastures at 20–40 g/kg DM are reported to increase polyunsaturated fatty acids in meat by reducing rumen biohydrogenation of unsaturated fatty acids. There is need to study the effect of *Desmanthus,* a tannin-containing legume, on performance and meat characteristics of grazing cattle.

Most of beef cattle in northern Australia are *Bos indicus* due to their ability to tolerate ticks, heat and poor-quality pasture. However, meat characteristics from these cattle is low due to low marbling. These breeds are crossed with *Bos taurus* to improve growth rate and meat characteristics of several composite breeds such as Belmont Red and NAPCO Composite. It is irrefutable that genetic make-up plays a significant role in beef fat content and FA profile.

- Several genes such as *SCD*, *FASN* and *FABP4* are reported to influence carcass fat traits in Korean and Japanese cattle as well as Australian temperate breeds such as Angus and Limousin. There is need to investigate the effect of these genes in northern Australian composite breeds.
- In addition, studies are required to determine finishing performance and carcass traits of northern Australian beef composite breeds backgrounded on newly introduced legume pastures, such as *Desmanthus*, to enable industry players to exploit them for greater economic gains.

## Figures and Tables

**Table 1:** Impact of supplements on beef cattle performance.

Pasture	Supplement	Outcome	Reference
*Urochloa decumbens*	Corn, Corn gluten, Soybean, Urea,	ADG up to 0.75 kg	[51]
*Urochloa decumbens* hay	Pure casein, urea and ammonia	Increase NDF digestion	[52]
*Urochloa decumbens* hay	Urea, ammonium sulphate and albumin	Increased DMI and	[53]
		NDF digestion	
*Urochloa brizantha*	Cottonseed meal, corn and urea	ADG of up to 0.3 kg	[54]
*Urochloa brizantha*	Soybean meal, urea and grain sorghum	ADG of up to 0.5 kg	[55]

ADG: average daily gain, NDF: neutral detergent fibre, DMI: dry matter intake.
